# Assessing the job preferences of senior medical students for mandatory service: a discrete choice experiment

**DOI:** 10.1017/S1463423624000252

**Published:** 2024-05-31

**Authors:** Buşra Tozduman, Melih Kaan Sözmen

**Affiliations:** 1 Dokuz Eylul University, Faculty of Medicine, Department of Public Health, Epidemiology Subsection, Izmir, Turkey; 2 Izmir Katip Çelebi University, Faculty of Medicine, Department of Public Health, Izmir, Turkey; 3 Department of Global Health and Population, Harvard T.H. Chan School of Public Health, Boston, MA 02115, USA

**Keywords:** discrete choice experiment, health workforce, physician, willingness to pay, workplace conditions

## Abstract

**Aim:**

To investigate the job preferences of senior medical students for mandatory service as general practitioners using discrete choice experiment.

**Introduction:**

Health workforce is directly associated with health service coverage and health outcomes. However, there is a global shortage of healthcare workers (HCWs) in rural areas. Discrete choice experiments can guide the policy and decision-makers to increase recruitment and retention of HCWs in remote and rural areas by determining their job preferences. The aim of this study is to investigate job preferences of senior medical students for mandatory service as general practitioners.

**Methods:**

This cross-sectional survey was conducted among 144 medical students. To estimate students’ preferences for different levels of job attributes, a mixed logit model was utilised. Simulations of job uptake rates and willingness to pay (WTP) estimates were computed.

**Findings:**

All attributes had an impact on the job preferences of students with the following order of priority: salary, workload, proximity to family/friends, working environment, facility and developmental status. For a normal workload and a workplace closed to family/friends which were the most valued attributes after salary, WTPs were 2818.8 Turkish lira (TRY) ($398.7) and 2287.5 TRY ($323.6), respectively. The preference weights of various job characteristics were modified by gender, the presence of a HCW parent and willingness to perform mandatory service. To recruit young physicians where they are most needed, monetary incentives appear to be the most efficient intervention. Non-pecuniary job characteristics also affected job preferences. Packages of both monetary and non-monetary incentives tailored to individual characteristics would be the most efficient approach.

## Introduction

Health workforce is regarded as a prerequisite for an effective and responsive health system and is also considered to be the key determinant of access to health services (Araújo and Maeda, 2013; Mohammadiaghdam et al., 2020). However, many countries are confronted with challenges in training, employing and deploying their workforce (Araújo and Maeda, 2013). There are also imbalances in the geographic distribution of healthcare workers (HCWs) within countries (World Health Organization, 2006). Globally, it is estimated that between 51% and 67% of the rural population has limited access to basic health care (WHO, 2019).

Turkey ranks last among the Organisation for Economic Co-operation and Development (OECD) countries in terms of the total number of physicians per capita (Ministry of Health, 2021). Additionally, there is a distribution disparity between rural and urban areas. In order to address this maldistribution, a number of financial and non-financial incentives have been introduced. Another intervention is the mandatory service requiring physicians to work in the public sector for a minimum of 10–20 months depending on their field of service, with restrictions on working in the private sector if not fulfilled. Despite these interventions, the density of physicians in Western Anatolia is twice that of the South-eastern Anatolia region (Ministry of Health, 2019). Considering the demographic and economic structure, health indicators of Turkey have not yet achieved the targeted level. In terms of life expectancy at birth, women’s and children’s health, control of communicable diseases such as tuberculosis and non-communicable diseases, and risk factors, Turkey ranks in the middle among world countries. The geographical region in which people live affects their access to health services in our country (Üner and Okyay, 2020).

HCWs’ employment decisions are a function of their preferences and expectations. Policies for recruitment and retention of HCWs in underserved areas should include a bundle of incentives. In order to assess HCWs’ preferences and predict the job uptake given a set of job characteristics, discrete choice experiments (DCEs) can be conducted (Araújo and Maeda, 2013).

DCE is a quantitative technique that assumes that goods and services can be described by their essential characteristics, and the value of a good or service can be derived from the combination of these characteristics (Ryan et al., 2001). In recent years, DCEs have become increasingly utilised in health economics, providing policy-makers with a basis for decision-making. For instance, DCEs have been employed to assess population preferences for vaccination (Adams et al., 2015; Dong et al., 2020; Lack et al., 2020); primary health care (Kleij et al., 2017; Lim et al., 2022); cancer, antenatal and newborn screening programmes (Lee et al., 2018; Vass et al., 2019); and tobacco control interventions (Regmi et al., 2018). Furthermore, this method has been employed to measure the preferences of health professionals and other stakeholders regarding the provision of health care (Hill et al., 2012, 2014; Leigh et al., 2020; Koopmanschap et al., 2010). Another common application of DCEs is to determine the job preferences of HCWs. DCEs provide quantitative information on the relative importance of job characteristics influencing HCWs’ preferences, as well as the trade-offs between these factors and changes in the probability of choices if levels within factors are changed (WHO, 2012).

## Aim

To examine (a) the job preferences and affecting individual characteristics (b) the salary students are willing to pay for desired working conditions and (c) to predict the impact of changes in job characteristics on the probability of choosing one job over another for mandatory service of senior medical students.

## Methods

This is a cross-sectional analytical study. The target population consisted of the last-grade medical students of a medical faculty (*n* = 144).

### Survey design

In order to ascertain the job characteristics (attributes) and levels, a literature review was conducted, and a semi-structured interview was carried out with 11 students. Six attributes, each with two to four levels were selected (Table 1). We opted to use the term ‘underdeveloped region’ instead of ‘rural area’ because in Turkey the urban–rural classification is based on population size (≤ 20 000 = rural; > 20 000 = urban) and does not accurately reflect the level of development.

**Table 1.** Job attributes, levels and definitions

Attributes	Levels	Definition
Facility	Ambulance command and control centre Community health centre Hospital Military medical centre	
Salary	6500 TL 8000 TL 9500 TL 11 000 TL	Total monthly income from working in the facility
Workload	Normal Heavy	There is enough time to do priority tasks during working hours There is barely enough time to do priority tasks during working hours
Working environment	Ideal Not ideal	Good communication between other HCWs, consultants, referrals and managers Poor communication between other HCWs, consultants, referrals and managers
Proximity to family/friends	Near Far	< 90 km > 90 km
Developmental status	Developed Underdeveloped	High standard of living, educational level, socioeconomic status Poor standard of living, educational level, socioeconomic status

HCWs = healthcare workers.

To construct efficient designs, Huber and Zwerina (1996) recommended utilising nonzero priors for parameter estimates. These prior values can be obtained from pilot studies. Uncertainty about priors should be taken into account as true parameter values can differ from assumed ones. The Bayesian design approach, introduced by Sándor and Wedel (2001), assumes a prior distribution of likely parameter values and optimises the design over that distribution (Sándor and Wedel, 2001).

Using a large number of attributes in DCEs can increase complexity and cognitive burden, contributing to an increased error variance. To simplify decision-making, some of the attributes’ levels can be held constant in every choice set (Jonker et al., 2018). The profiles in such a choice set are called partial profiles. Kessels et al. constructed D-optimal partial profile designs using a Bayesian design algorithm that integrates the D-optimality criterion over a prior distribution of likely parameter values and implemented it in statistical software package JMP (Kessels et al., 2011).

We used JMP Pro 14 (SAS Institute, Cary, NC) to generate 12 choice sets, each consisting of 2 profiles for pilot study. At least two attributes were held constant in each choice set. This approach is common in DCEs in health economics, which typically have 16 or fewer choice sets with 4–6 attributes (de Bekker-Grob et al., 2012). Since the participants are physician candidates who have mandatory service obligations, ‘opt out’ or ‘status quo’ alternatives were not included in the design. A pilot study was conducted with 11 students to determine the prior values. Based on this prior information, the final choice design was constructed. Three different versions of the choice design were generated to improve design efficiency. Each version contained 12 choice sets and 24 profiles with different combinations of attribute levels. Additionally, to identify respondents whose preferences violated common rationality, a choice set was inserted between the 12 pairs. This choice set had the same levels for all attributes except for salary. The job offering a higher salary was expected to be chosen. This choice set was not used in the main regression analysis.

The DCE tool also included questions on respondents’ socio-demographic characteristics and attitudes towards mandatory service. All participants received one version of questionnaire online and were asked to select one of the two job scenarios from each choice set. Data were collected during January–March 2021.

### Respondents

Sample size was calculated as 84 using Johnson and Orme’s method (Johnson and Orme, 2003; Orme, 1998). We aimed to reach all last-grade medical students of the faculty without selecting a sample to conduct subgroup analysis.

### Data analysis

All data from the DCE questionnaires were stored using Microsoft Excel 2016 (Microsoft Corporation, USA). The general characteristics of the students were summarised as median (min–max) or frequencies and percentages. Salary was coded as a continuous variable, and other attributes were dummy coded, with 1 representing their presence in each profile and 0 representing their absence. Following this, the mixed logit (MXL) model was used to estimate participants’ preferences for the different levels of the job attributes using Stata® 15.0 (Stata Corporation, USA) with user-written codes (Hole, 2013). The MXL model accounts for the panel data, allowing for multiple observations from each respondent (Hauber et al., 2016). Furthermore, the model accommodates heterogeneity in preferences across the sample by treating coefficients as random. In our study, the salary attribute was specified as fixed to facilitate the calculation of willingness to pay (WTP), while all other attributes were specified as having a random component. To explain the sources of heterogeneity, interactions of gender, hometown, income, having a HCW parent and willingness to perform mandatory service with attributes were included. The model presented in Table 2 includes the interaction terms which were statistically significant. The changes in the probability of choices were calculated using Hole’s ‘mixlpred’ command, in which the levels of attributes were altered. Additionally, the monetary value of attribute levels, namely, WTP and confidence intervals, was estimated using ‘wtp’ command in Stata (Hole, 2013).

**Table 2.** The general characteristics for last-grade medical students

	Median (min–max)
Age	24 (22–34) years
	*n* (%)
Gender (female)	60 (56.1%)
Marital status (single)	107
Hometown	
City	65 (60.8%)
County	35 (32.7%)
Village	7 (6.5%)
Income	
Less than expenditure	23 (21.5%)
Equal to expenditure	57 (53.3%)
More than expenditure	27 (25.2%)
HCW parent (yes)	22 (20.6%)
Willingness to perform compulsory service	
Yes	71 (66.4%)
No/Not sure	36 (33.6%)
Planning to pursue specialisation	
Yes	102 (95.3%)
No	0
Not sure	5 (4.7%)

HCW = healthcare worker.

### External validity

The questionnaire was also delivered to students from other medical faculties. We performed a 1:1 propensity score matching in IBM SPSS Version 25.0 to include students from other faculties who best matched. The propensity score was calculated based on participants’ gender, age, marital status, income, hometown, having a HCW parent, willingness to perform mandatory service and intention to pursue specialisation. To assess external validity, the results from two groups were compared by calculating the Kappa coefficient (Parady et al., 2021).

## Findings

Of the 144 medical students who were recruited, 107 (%74.3) respondents completed the questionnaire. A total of five (3.5%) respondents failed the rationality test. The estimated models with and without these respondents did not differ significantly, and thus, these respondents were retained in the main analysis. None of the students exhibited a dominant preference, indicating that they all trade off attribute levels.

Table 2 presents the characteristics of the respondents. 56.1% of the participants were female with a median age of 24 years. All of the respondents were single. Only 6.5% of medical students had a rural background (ie, had grown up in a village). Approximately half of the students reported their income status as income equal to expenditure. 20.6% of the students had a HCW parent. While 66.4% of the participants indicated that they would perform mandatory service as general practitioners, almost all of them were planning to pursue specialisation in the upcoming years.

Table 3 presents the MXL model which includes main effect and interaction terms. The MXL model indicates that students exhibited a preference for employment in a hospital or community health centre (CHC) over a military medical centre [β (S.E.) = 0.71 (0.23); *P* < 0.01, β (S.E.) = 0.88 (0.23); *P* < 0.001, respectively]. A higher salary [β (S.E.) = 6 × 10^−4^ (6 × 10^−5^) per Turkish lira (TRY); *P* < 0.001], normal workload [β (S.E.) = 1.81 (0.22); *P* < 0.001] and an ideal working environment [β (S.E.) = 1.27 (0.19); *P* < 0.001] significantly increased the likelihood a job being selected. Students demonstrated a preference for facilities located in developed regions and closer to their family/friends [β (S.E.) = 0.6 (0.16); *P* < 0.001, β (S.E.) = 1.47 (0.19); *P* < 0.001, respectively].

**Table 3.** Estimation results from the mixed logit model

	Mixed logit model		Mixed logit with interaction terms	
	β (S.E.)	β std. (S.E.)	β (S.E.)	β std. (S.E.)
Salary	6 × 10^−4^ (6 × 10^−5^)^***^	–	5 × 10^−4^ (9 × 10^−5^)^***^	–
Facility (base: military medical centre)				
Ambulance command and control centre	−0.04 (0.23)	1.03 (0.23)^***^	0.003 (0.24)	1.04 (0.23)^***^
Hospital	0.71 (0.23)^**^	0.25 (0.42)	0.53 (0.25)*	0.09 (0.43)
CHC	0.88 (0.23)^***^	0.31 (0.81)	0.12 (0.37)	−0.21 (0.52)
Workload (base: heavy)	1.81 (0.22)^***^	1.34 (0.22)^***^	1.91 (0.24)^***^	1.56 (0.24)^***^
Working environment (base: not ideal)	1.27 (0.19)^***^	1.31 (0.20)^***^	1.32 (0.19)^***^	1.38 (0.21)^***^
Proximity to family/friends (base: far)	1.47 (0.19)^***^	0.76 (0.24)^**^	1.22 (0.3)^***^	0.76 (0.23)^***^
Developmental status (base: underdeveloped)	0.6 (0.16)^***^	0.47 (0.28)	1.6 (0.32)^***^	0.09 (0.6)
Developmental status *Gender (ref: male)			−0.71 (0.26)^**^	–
Hospital* HCW parent (ref: have a HCW parent)			1.3 (0.49)^**^	–
CHC* HCW parent			1.29 (0.46)^**^	–
CHC* willing to perform compulsory service (ref: yes)			0.89 (0.4)^*^	–
Proximity to family/friends *Willing to perform compulsory service			0.57 (0.35)	–
Developmental status *willing to perform compulsory service			−0.84 (0.32)^**^	–
Salary*willing to perform compulsory service			0.0003 (0.0001)^**^	–
Observations	2568		2568	
Log-likelihood (null)/(model)	−675.6814/−632.2966		−655.1219/−606.9331	
LR χ^2^, Prob > chi2	86.77, < 0.001		96.38, < 0.001	
AIC/BIC	1294.593/1382.356		1257.866/1386.586	
McFadden’s R^2^	0.064		0.073	

CHC = community health centre; HCW = healthcare worker; LR, log-likelihood ratio; AIC: akaike information criterion; BIC: Bayesian information criterion.

* *P* < 0.05.

** *P* < 0.01.

*** *P* < 0.001.

The regression results indicate the presence of significant unobserved preference heterogeneity between respondents (as evidenced by the significant standard deviation of the random attribute coefficients). To elucidate the sources of this heterogeneity, a model was also estimated in which participant-specific characteristics were permitted to interact with job attributes. The log-likelihood ratio test [χ2 (df:7) = 50.727, *P* < 0.001] rejected the null hypothesis that the regression parameters for the MXL model and the MXL model with interactions are equal at 0.5% significance level, indicating that the model fit has improved with an R^2^ of 0.073. The results of the MXL model with interactions suggest that males place a greater value on the development status of the region more than females. Those with a parent employed in the healthcare sector exhibit a stronger preference for working at a hospital or CHC. There is a greater inclination to work at a CHC and for a higher salary among students willing to perform mandatory service, while others value the development status of the region more. The MXL model with interaction terms also yielded significant derived standard deviations for workload, working environment and proximity to family/friends indicating the existence of unobserved heterogeneity for these attributes.

Table 4 presents the WTP values, which can be described as the salary students would be willing to sacrifice for improvements in job characteristics. The respondents indicated a WTP of 1102.6 TRY (95% CI, 385.1–1820.1 TRY) to work at a hospital and 1372.9 TRY (95% CI, 645–2100.7 TRY) at CHC, 2818.8 TRY (95% CI, 2160.3–3477.3 TRY) for a normal workload, 1968.5 TRY (95% CI, 1401.0–2536 TRY) for an ideal working environment, 2287.5 TRY (95% CI, 1752.8–2822.2 TRY) for a workplace nearer to family/friends and 930.2 TRY (95% CI, 486.5–1373.8 TRY) for a job located in a developed region (the exchange rate from February 2021 of US$1 = 7.07 TRY, 1 Euro = 8.56 TRY) (TCMB Central Bank of the Republic of Turkey Currency Exchange Rates., 2021).

**Table 4.** Willingness to pay estimates for job attributes

	WTP	(%95 CI)
Facility (base: military medical centre)		
Ambulance command and control centre	−62.7 TL	(−773.1)–647.7
Hospital	1102.6 TL	385.1–1820.1
CHC	1372.9 TL	645–2100.7
Workload (base: heavy)	2818.8 TL	2160.3–3477.3
Working environment (base: not ideal)	1968.5 TL	1401.0–2536
Proximity to family/friends (base: far)	2287.5 TL	1752.8–2822.2
Developmental status (base: underdeveloped)	930.2 TL	486.5–1373.8

CHC = community health centre.

**Figure 1.**
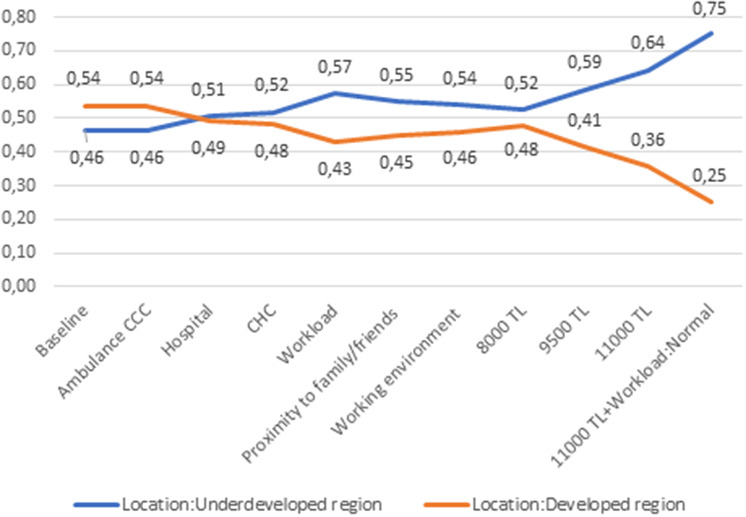
Probabilities of taking a job located in a developed region versus underdeveloped region with changing job conditions

Figure 1 illustrates the varying probabilities of accepting a position in a developed versus an underdeveloped region, contingent upon the specific job conditions. *Ceteris paribus*, the probability of accepting a position in an underdeveloped region is 46%, whereas the probability of accepting a position in a developed region is 54%. The probability of choosing a hospital in an underdeveloped region is 51% and that of choosing a CHC in an underdeveloped region is 52%, in comparison to a military medical centre in a developed region. The probability of selecting a job in an underdeveloped region would increase to 57%, 55% and 56%, respectively, if the workload were to be improved, if the location were to be closer to family or friends and if the working environment were to be more favourable. An increase in salary from 6500 TRY to 8000 TRY would result in a 52% probability of choosing an underdeveloped region. The model predicted that introduction of a normal workload with 11 000 TRY per month salary rather than heavy workload with 6500 TRY per month salary would increase the proportion of students opting for a job in an underdeveloped region to 75%.

Indicating the external validity, kappa coefficients for all choice sets were significant, and total kappa score was 0.819 (*P* < 0.001).

## Discussion

This DCE has elicited preferences for job attributes among the senior medical students. All six attributes significantly affected the students’ job choices. They preferred to work at a hospital or CHC, closer to family/friends in a developed region with a higher salary, a normal workload and an ideal working environment. The MXL model estimates revealed the existence of preference heterogeneity in workload, working environment and proximity to family/friends. The gender of the respondents, the presence of a HCW parent and the willingness to perform mandatory service were found to affect the preference weights of certain job characteristics.

Similar with other studies, salary was found to be the most important factor influencing job preferences (Karyani et al., 2020; T. Liu et al., 2019; Vujicic et al., 2010). Additionally, students who were willing to perform mandatory service demonstrated a stronger preference for higher salaries. Given that the majority of students expressed a desire to pursue specialisation training, it can be hypothesised that the motivation for performing mandatory service may be financial. In 2017, the Turkish Ministry of Health conducted a survey to examine the job satisfaction of healthcare staff. The findings of this study indicated that salary is the most important factor influencing job satisfaction and that it is one of the HCWs’ strongest demands to be regulated in the healthcare system (Health Personnel Satisfaction Survey, 2017). In another DCE conducted in Turkey, salary was ranked as the second most important attribute among general practitioners (İşlek and Şahin, 2023). According to these findings, providing economic incentives should be a priority.

Workload was the most significant non-monetary attribute influencing job preferences. This finding aligns with previous DCEs, which have demonstrated that HCWs are reluctant to accept heavy workloads and value having adequate leisure time (Rafiei et al., 2015; Scott et al., 2020; Sivey et al., 2012). In the Turkish Healthcare staff job satisfaction survey, half of the respondents claimed to have a heavy workload (Health Personnel Satisfaction Survey, 2017). Islek et al. reported that workload has a significant effect on the job preferences of physicians under the age of 35 years (İşlek and Şahin, 2023). It is suggested that young physicians increasingly prioritise work–life balance and believe that they do not have to work as much as previous generations to make a living (Bao and Huang, 2021; Harding et al., 2016; Matthews et al., 2012). Furthermore, the majority of respondents indicated that they planned to pursue specialisation. A normal workload was perceived to mean more free time to study for the residency examination for them. This result is consistent with DCE studies conducted in China, Mozambique and Kenya which found that career development and training were regarded as important attributes of job preferences (Honda and Vio, 2015; S. Liu et al., 2018; Takemura et al., 2016).

The proposed study showed that proximity to family/friends of job also had a substantial effect on job preferences, in line with the studies carried out in Australia and Canada (Harding et al., 2016; Matthews et al., 2012; Szafran et al., 2001). The lack of social support causes depression and burnout among physicians (Kuhn and Flanagan, 2016). Furthermore, only 4% of the participants stated that they intend to complete their mandatory service. Since this is a relatively short-term and temporary period for the majority of the participants, they might take into account the housing conditions and prefer closer workplaces to their family and friends.

Similar to previously reported DCEs, our respondents had a preference for an ideal working environment (Awases et al., 2004; Zurn et al., 2004). This finding also concurs with studies that have identified concerns among medical students regarding their professional competence (Aker and Mıdık, 2020; Ergin et al., 2016; Yalçinoğlu et al., 2012). It can be reasonably assumed that newly graduated physicians will expect their employers to provide them with a supportive management structure, as well as the opportunity to consult, refer and collaborate with specialists and more experienced colleagues.

Although the facility had a significant effect on job preference, there was a substantial heterogeneity among the respondents. According to our findings, students with an HCW parent were more likely to work at CHC and hospital. Students who were willing to perform mandatory service were also more likely to work at CHC. The majority of the health workforce is employed in hospitals and CHCs in our healthcare system. All of the medical faculties include rotations to these facilities in their training programme. As a result, students are expected to be more familiar with the working conditions of these facilities. In hospitals, general practitioners are frequently employed in emergency services and are required to cope with the stress associated with night shifts (Ağapınar and Şahin, 2014). Furthermore, there are no on-calls or night shifts in CHCs, and the risk of malpractice is relatively low. Consequently, CHCs have a higher preference weight than hospitals.

The developmental status of the work location was also valued by the participants. This finding is consistent with other researches that suggest that HCWs tend to prefer centrally located jobs (İşlek, 2021; İşlek and Şahin, 2023; Kolstad, 2011; S. Liu et al., 2018; Smitz et al., 2016). Rural and remote areas are perceived as less desirable due to limited educational opportunities for children, inadequate infrastructure (communication and transportation) and limited career options for spouses (Lehmann et al., 2008; S. Liu et al., 2018). Some regulations have been enacted to address this issue. For instance, the duration of mandatory service and the amount of additional payments vary depending on the developmental status of the area (Basic Health Services Law, 1987). Nevertheless, these incentives are insufficient to address the shortage of physicians in underdeveloped areas in our country. The MXL model with interaction terms indicated that males and students who were unwilling to perform mandatory service valued developmental status more than others. As there are differences across the studies, gender is not a consistent predictor for choosing a rural post (Isaac et al., 2015; Jones et al., 2009; Kim et al., 2020; King et al., 2016; Playford et al., 2014; Puddey et al., 2014). Further investigation is required to ascertain the extent to which other factors contribute to this association.

### Conclusion

Our study indicates that monetary incentives are crucial to recruiting newly graduated physicians where they are mostly needed. Bundles of both monetary and non-monetary incentives, tailored to individual characteristics, would be more efficient than a single intervention.

In our country, primary healthcare services, catering to both the community and individuals, are primarily provided by general practitioners. Family medicine positions were not included in this study due to their contractual nature. However, students expressed a preference for working in primary healthcare institutions. Nevertheless, nearly all participants expressed a keenness for specialisation. This tendency could precipitate a rapid turnover of physicians, leading to service disruptions. The results of this study offer valuable insights for crafting incentive schemes aimed at attracting and retaining physicians in primary healthcare settings. Similar study frameworks could be devised for specialist physicians and other healthcare professionals across various institutions (family medicine/CHC/provincial health directorate) and fields (communicable diseases/environmental health/vaccination/non-communicable diseases/reproductive health/occupational health). Furthermore, the cost-effectiveness of different incentive schemes can be calculated in future works.

This is the first study using DCE methodology to investigate the job preferences of medical students in our country. Another strength is our utilisation of a pilot survey to create prior values for the coefficients in our experimental design. To control the capability of accurate prediction of our model, we conducted an external validity analysis. It is assumed that respondents apply compensatory decision rules in DCEs. Hence, dominant preferences have been checked.

This study has several notable limitations. Firstly, since this is a single-centre research, the results cannot be generalised to the whole country. Secondly, due to the hypothetical nature of DCEs, there may be disparities between revealed and stated preferences. Thirdly, DCEs imply a certain degree of simplification to limit the number of job attributes and levels. Therefore, many other job characteristics that are likely to affect a HCW’s employment decisions may have been overlooked. It is recommended that policy-makers should validate DCEs’ findings before implementing a specific bundle of interventions (Araújo and Maeda, 2013).

## Data Availability

The data that support the findings of this study are available on request from the corresponding author.
